# Formative Research to Design SMS Messages to Increase Breast Cancer Screening Uptake in Argentina

**DOI:** 10.1200/GO.22.32000

**Published:** 2022-05-05

**Authors:** Victoria Sanchez Antelo, Melisa Paolino, Paula Frejdkes, Silvina Correa, Anabel Furia, Graciela Lopez de Degani, Silvina Arrossi

**Affiliations:** ^1^ Centro de Estudios de Estado y Sociedad/Consejo Nacional de Investigaciones Científicas y Técnicas (CEDES/CONICET), Buenos Aires, Argentina; ^2^ Centro de Estudios de Estado y Sociedad, Buenos Aires, Argentina; ^3^ Ministerio de Salud de Santa Fe, Santa Fe, Argentina; ^4^ Agencia de Control del Cáncer—Ministerio de Salud de Santa Fe, Santa Fe, Argentina; ^5^ Agencia del Control del Cáncer—Ministerio de Salud de Santa Fe, Santa Fe, Argentina

## Abstract

There is a need for interventions to promote breast cancer screening among high-risk population. This formative research is part of a pragmatic randomized trial being conducted in Santa Fe, Argentina, in which we are evaluating whether SMS messages sent to women aged 50-69 increases breast cancer screening uptake. Here, we describe the formative results used to design the content of the SMS messages to be tested in the trial.

**METHODS:**

We conducted four on-line focus groups with women aged 50+ (n = 14). Participatory techniques were used to debate the advantages and disadvantages of different options for the five structural elements -i.e., greeting, recipient, sender, message's topic, and closing line-of the SMS message. We openly coded the discussions for agreements and preferences regarding the SMS message content.

**RESULTS:**

SMS messages as reminders to increase breast cancer screening were highly accepted. Women argued that the greeting line should provide clear information about the topic of the SMS message (eg, Health information). SMS messages should also include the woman's name, because this inclusion would be an indicator that it was a personal SMS message. Most women considered that the sender of the SMS messages should be a health institution as this would legitimize the content. Regarding the topic of the message, women preferred an imperative tone and they mentioned that the SMS message should include information about how to get a mammogram (eg, “Women aged 50-69 should have a mammogram done every two years. Do you have an appointment? WhatsApp to xxxxx”). The closing line should encourage women to get a mammogram (eg, “Ask for your appointment now! It is important!”).

**CONCLUSION:**

Our findings have some implications for the design of mHealth interventions targeted at improving breast cancer screening. A personalized SMS could be a good way of inviting women, although its content must be carefully designed to provide clear information about how to get a mammogram.

